# Chronic dietary supplementation with soy protein improves muscle function in rats

**DOI:** 10.1371/journal.pone.0189246

**Published:** 2017-12-07

**Authors:** Ramzi J. Khairallah, Karen M. O’Shea, Christopher W. Ward, Dustie N. Butteiger, Ratna Mukherjea, Elaine S. Krul

**Affiliations:** 1 Myologica, LLC, New Market, MD, United States of America; 2 Department of Orthopedics, University of Maryland School of Medicine, Baltimore, MD, United States of America; 3 DuPont Nutrition & Health, St. Louis, MO, United States of America; West Virginia University School of Medicine, UNITED STATES

## Abstract

Athletes as well as elderly or hospitalized patients use dietary protein supplementation to maintain or grow skeletal muscle. It is recognized that high quality protein is needed for muscle accretion, and can be obtained from both animal and plant-based sources. There is interest to understand whether these sources differ in their ability to maintain or stimulate muscle growth and function. In this study, baseline muscle performance was assessed in 50 adult Sprague-Dawley rats after which they were assigned to one of five semi-purified “Western” diets (n = 10/group) differing only in protein source, namely 19 kcal% protein from either milk protein isolate (MPI), whey protein isolate (WPI), soy protein isolate (SPI), soy protein concentrate (SPC) or enzyme-treated soy protein (SPE). The diets were fed for 8 weeks at which point muscle performance testing was repeated and tissues were collected for analysis. There was no significant difference in food consumption or body weights over time between the diet groups nor were there differences in terminal organ and muscle weights or in serum lipids, creatinine or myostatin. Compared with MPI-fed rats, rats fed WPI and SPC displayed a greater maximum rate of contraction using the *in vivo* measure of muscle performance (p<0.05) with increases ranging from 13.3–27.5% and 22.8–29.5%, respectively at 60, 80, 100 and 150 Hz. When the maximum force was normalized to body weight, SPC-fed rats displayed increased force compared to MPI (p<0.05), whereas when normalized to gastrocnemius weight, WPI-fed rats displayed increased force compared to MPI (p<0.05). There was no difference between groups using *in situ* muscle performance. In conclusion, soy protein consumption, in high-fat diet, resulted in muscle function comparable to whey protein and improved compared to milk protein. The benefits seen with soy or whey protein were independent of changes in muscle mass or fiber cross-sectional area.

## Introduction

Skeletal muscle accounts for a significant proportion of the body’s total lean mass. Beyond its central role in physical movement, energy metabolism and temperature regulation, skeletal muscle is the largest protein/amino acid source in the body and a reservoir for water, minerals, and vitamins. While it is well accepted that physical activity and an adequate intake of high quality protein is key to building muscle mass, [1] the ingestion of high quality protein is a necessary anabolic driver to maintain muscle mass in the absence of strenuous exercise [2].

Dietary protein supplementation is widely used in recreation and for professional athletes, as well as in at-risk populations (i.e., aged, ill) where high quality protein is needed to optimize the maintenance or growth of skeletal muscle. Given that high quality dietary protein is available from both animal and plant based sources, there has been long standing interest in whether these sources differ in their ability to maintain or stimulate muscle growth.

Chan et al. [3] reported no differences in changes in appendicular skeletal muscle mass or physical performance in a 4 year follow-up study in a Chinese population consuming a habitual diet containing different amounts of total protein. However, this group noted that a higher plant-based protein diet (> 0.72 g/kg body weight) was associated with reduced muscle loss compared with those consuming a lower amount of plant-based protein diet (< 0.4 g/kg body weight)(P_trend_ = 0.025). Similarly, in an elderly cohort of Japanese women, there was a reduced decline over 4 years in knee extension strength in women who consumed more soy products or green and yellow vegetables [4]. In contrast, in a small study of women in Finland, habitual consumption of a vegetarian diet was associated with a lower muscle mass index compared with an omnivorous diet [5]; however, physical function was not assessed in that study.

It is recognized that skeletal muscle mass does not necessarily predict muscle strength or specific force, since metabolic conditions [6] or age [7] can affect these functional properties. In a cross-sectional study of an elderly Quebec community dwelling population, animal protein intake was positively associated with HOMA-IR score, a measure of insulin resistance, whereas a higher plant-based protein intake had an opposite association [8]. However these investigators noted a negative association between muscle mass index (based on height) and plant protein intake [8].

Given that the impact of dietary protein can influence both the mass and function of skeletal muscle, studies investigating the long-term diet effects should include an evaluation of both mass and physical function. Acute studies, while useful in assessing the direct effect of dietary protein on muscle protein synthesis, cannot predict the long-term impact on muscle structure and function nor overall metabolic effects that can impact muscle physiologic processes.

The objective of this study was to assess the impact of different dietary protein sources, animal and plant-based, in the context of a typical “Western” diet on muscle morphology, size and function in a sedentary rat model. Two different dairy sources of protein (milk protein isolate and whey protein isolate) and three differently processed soy protein products (soy protein isolate, soy protein concentrate and a novel enzyme-treated soy protein isolate) were evaluated. The rat model is a well-accepted model used in research on muscle metabolism and function. Using animal models, which are genetically homogeneous and can be maintained in a controlled environment, allows one to compare the effects of different dietary components on muscle physiology with relatively few confounding factors. It would be anticipated that any differential effects would be more readily detected in the rat model compared with human feeding studies.

## Methods

### Animals and diets

Adult male Sprague-Dawley® rats weighing 473 ± 3 g from Charles River were maintained in a temperature controlled room, with a 12-h light/dark cycle and allowed free access to a standard laboratory rat chow (Harlan Teklab Global 16% Protein Rodent Diet #2016C (Harlan, Madison, WI) and water. On day 19 of the study, the rats were assigned to one of five semi-purified “Western” diets (n = 10/diet group) containing 22.5(g%) protein as Milk Protein Isolate (MPI), Whey Protein Isolate (WPI), Soy Protein Isolate (SPI), Soy Protein Concentrate (SPC), or Enzyme-treated Soy Protein (SPE) for a total of 8 weeks. The diets were prepared by Research Diets, Inc., (New Brunswick, NJ USA) and provided 19%, 45% and 36% of energy from protein, carbohydrates and fat, respectively (Table 1). The study protocol was reviewed and approved by the Institutional Animal Care and Use Committee of Spring Valley Laboratories, Inc. and conducted at Spring Valley Laboratories, Inc. The animals were cared for according to the NIH Guide for the Care and Use of Laboratory Animals.

**Table 1:** Composition of experimental diets.

	Diets				
Ingredient	MPI	WPI	SPI	SPC	SPE
	gm				
Milk Protein Isolate^1^	215.4	-	-	-	
Whey Protein Isolate^2^	-	199.1	-	-	
Soy Protein Isolate^3^	-	-	196.8	-	
Soy Protein Concentrate^4^	-	-	-	223.1	
Soy Protein Isolate, enzyme-treated^5^					200.9
DL-Methionine	3	3	3	3	3
Corn starch	240	240	240	240	240
Maltodextrin 10	75	75	75	75	75
Sucrose	100	100	100	100	100
Cellulose, BW200	50	50	50	50	50
Cocoa Butter	37.5	37.5	37.5	37.5	37.5
Linseed Oil	4.5	4.5	4.5	4.5	4.5
Palm Oil	52.5	52.5	52.5	52.5	52.5
Safflower Oil	28.5	28.5	28.5	28.5	28.5
Sunflower Oil, High Oleic	27	27	27	27	27
tBHQ	0.03	0.03	0.03	0.03	0.03
Salts, S10026	10	10	10	10	10
DiCalcium Phosphate	13	13	13	13	13
Calcium Carbonate	5.5	5.5	5.5	5.5	5.5
Potassium Citrate, 1 H_2_O	16.5	16.5	16.5	16.5	16.5
Vitamin Mix, V13401	10	10	10	10	10
Vitamin E Acetate. 50%	0.13	0.13	0.13	0.13	0.13
Choline Bitartrate	2	2	2	2	2
Protein kcal%	19	19	19	19	19
Carbohydrate kcal%	44.6	44.6	44.6	44.6	44.6
Fat kcal%	36.3	36.3	36.3	36.3	36.3

^1^MPI-85, Idaho Milk Products

^2^WPI, Whey Protein Isolate, Hilmar™ 9410

^3^SUPRO®760, Soy Protein Isolate, DuPont Nutrition & Health

^4^ALPHA®5800, Soy Protein Concentrate, DuPont Nutrition & Health

^5^Soy Protein Isolate, Enzyme-Treated, DuPont Nutrition & Health (non-commercial)

Animals in the current study were matched for weight and baseline muscle performance prior to assignment to the diet groups. Food consumption was monitored for the duration of dietary treatment. The rats were housed 1/cage, and the food in each cage was weighed on a weekly basis. Animals underwent muscle performance testing and were sacrificed at 8 weeks. Animals were anesthetized using isoflurane and sacrificed by exsanguination. Blood was taken by cardiac puncture and plasma collected. Tissues, including left gastrocnemius muscle, extensor digitorum longus (EDL), tibialis anterior, soleus, quadriceps, triceps, heart, liver, suprarenal fat pad, retroperitoneal fat pad, and epididymal fat pad, were excised, rinsed with PBS, weighed, and immediately frozen in liquid nitrogen. Right gastrocnemius muscles were excised, embedded in cryomatrix, and frozen in 2-methylbutane cooled by liquid nitrogen for histological analysis.

### Serum biochemical analyses

Creatinine and total cholesterol were measured using an enzymatic, colorimetric assay. Triglycerides were measured using a glycerol blanked, enzymatic, colorimetric assay. Testing was performed on the c501 module of the Roche Cobas 6000 analyzer. Reagents for creatinine, total cholesterol and triglycerides are manufactured by Roche Diagnostics. Myostatin was measured using an ELISA according to the manufacturer’s instructions (R&D Systems Inc., Minneapolis, MN). These measures were conducted at the Core Laboratory for Clinical Studies at Washington University School of Medicine in St. Louis.

### Muscle performance assay

Muscle performance was measured *in vivo* with a 305C muscle lever system (Aurora Scientific Inc., Aurora, CAN). Anesthesia in the rat was accomplished via inhalation (~5% isoflurane, or to effect), and maintained via nose-cone (~2% isofluorane, or to effect, ensuring proper anesthesia without respiratory distress). Hair was removed from the lower leg by application of depilatory cream for 3 minutes, followed by thorough rinsing with physiological buffer. The leg was then wiped with a 5% povidone-iodine solution followed by 70% isopropyl alcohol. The knee was isolated using a pin through the tibial head and the foot firmly fixed to a footplate on the motor shaft. Contractions were elicited by percutaneous electrical stimulation of the sciatic nerve and plantarflexor force production measured. Optimal isometric twitch torque was determined by increasing the current with a minimum of 30 s between each contraction to avoid fatigue. For force frequency determination, a series of stimulations were then performed at increasing frequency of stimulation (0.2 ms pulse, 250 ms train duration): 1, 10, 20, 40, 60, 80, 100, 150 Hz, followed by a final stimulation at 1 Hz. The muscle was then allowed to rest for 5 min. For optimal isometric force determination, a series of tetanic stimulations (80 Hz, 0.2 ms pulse, 250 ms duration) were delivered at 0.3 Hz frequency for 5 minutes to determine fatigue. Following the measurements, the foot was released and the pin removed. After baseline *in vivo* testing, each rat recovered in a separate clean cage prior to returning to its living chamber. At the end of the study (8 weeks post-dietary assignment), the rats underwent *in vivo* and *in situ* muscle performance testing and were then sacrificed.

Data were analyzed using Aurora Scientific 615A Dynamic Muscle Analysis Software Suite in high-throughput mode. The software automatically determines a baseline, a maximum and a minimum. The baseline is then subtracted from the peak force to give maximal active force. The start of the stimulus is automatically registered and each data trace is fitted to determine a maximum and minimum dx/dt, equivalent to the maximal rate of contraction and relaxation respectively. Each data file was manually inspected to ensure that cursors and fits were assigned properly and corrected when necessary.

Data were then grouped and means and standard errors calculated. Force was normalized to animal body weight and muscle weight when available. Force-frequency response was analyzed by normalizing the force produced at a given stimulation frequency to the force produced at 150 Hz stimulation. Tetanic-to-twitch ratio was calculated by dividing the force produced by a 150 Hz stimulation by the force produce by a single twitch. Data were compared over time by calculating the percent change using standard equations.

### Histology and muscle fiber typing

Serial sections (10 μm thickness) of cryo-preserved muscle were taken perpendicular to the fiber axis. Multiple slices (5–10) were taken at different portions of the muscle. The slices were then fixed in ice-cold paraformaldehyde and kept at -80°C until further use.

For cross-sectional area determination, fixed sections from the mid-belly of the muscle of interest were stained with wheat germ agglutinin conjugated to a fluorophore to visualize cell membranes. Sections were digitized using fluorescent microscopy, cell boundaries were traced using predictive software, and cross-sectional area was determined via unbiased automated measurements. For muscle fiber type determination, histological slices were taken from the mid-belly of the soleus and the gastrocnemius muscle. The fixed tissue sections were then blocked using Tris-buffered saline supplemented with 4% bovine serum albumin, 0.01% Triton X-100 and 10μg/ml Fab fragments for 1 h at room temperature. The slide was then washed with PBS and covered with a primary antibody against either MyHC-I, MyHC-IIa, MyHC-IIb, or Pax7 (for satellite cells) at the recommended dilution and incubated overnight at 4°C. The slide was then washed with PBS and the appropriate secondary antibody added for 1 h at room temperature. The slide was washed again in PBS, covered in mounting solution, and a coverslip used to seal the tissue section for fluorescence microscopy measures. Fluorescently-labeled tissue sections were digitized using a fluorescent microscope. Images were then analyzed for number of cells using standard counting software. Number of cells per volume were extrapolated from the section volume and the muscle weight.

### Statistical analysis

Statistical analysis was performed using SigmaPlot 11.0. Muscle performance data were analyzed using a two-way repeated measures ANOVA for stimulation frequency and dietary treatment with an α = 0.05 using the MPI group as the control group. Post hoc analysis was performed using a Holm-Sidak test for pairwise comparisons. A one-way ANOVA was used for assessing differences due to dietary treatment, with Holm-Sidak tests for post hoc analysis.

## Results

### Baseline measures

#### Body weight and muscle performance

**Fig 1:**
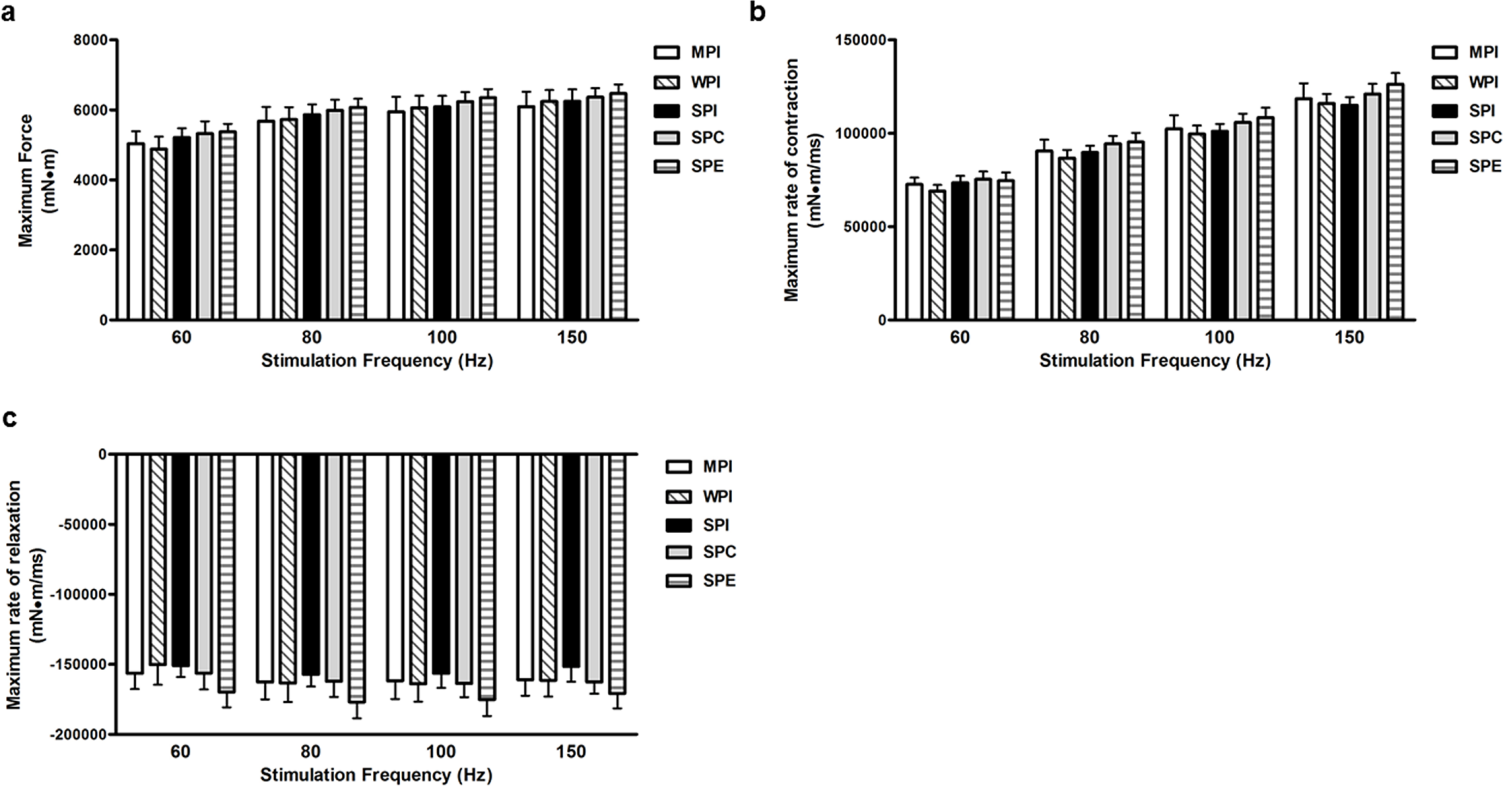
Baseline *in vivo* muscle performance. Values are means ± SEM. **a.** Maximum force *in vivo* in the plantarflexor at baseline. **b.** Maximum rate of contraction *in vivo* in the plantarflexor at baseline. **c.** Maximum rate of relaxation *in vivo* in the plantarflexor at baseline.

The animals were assigned to one of the 5 diet groups with no differences among the 5 groups for body weight at baseline. Given that animals were assigned to groups following force measurement, at baseline, there were no differences across groups for muscle performance as assessed by maximal isometric force (S1A Fig, maximum force, maximum rate of contraction, and maximum rate of relaxation(Fig 1A–1C).

### End-point biochemical and morphological measures

#### Food consumption and body weight

There were no significant differences in food consumption (data not shown) or body weight over time (S1 Table) between the different diet groups.

#### Serum creatinine and lipids

There were no significant differences in serum creatinine or serum lipids between diet groups at the end of the study, however, there was a trend towards lower serum lipids in the soy protein isolate and concentrate groups compared with the milk protein isolate group (S1 Table).

#### Serum myostatin

There were no significant differences in serum myostatin or between diet groups at the end of the study (S2 Table).

#### Organ and muscle weights

There were no significant differences in organ and muscle weights between diet groups at the end of the study (S3 Table).

### End-point measures of muscle performance *in vivo*

#### Contraction and relaxation kinetics

**Fig 2:**
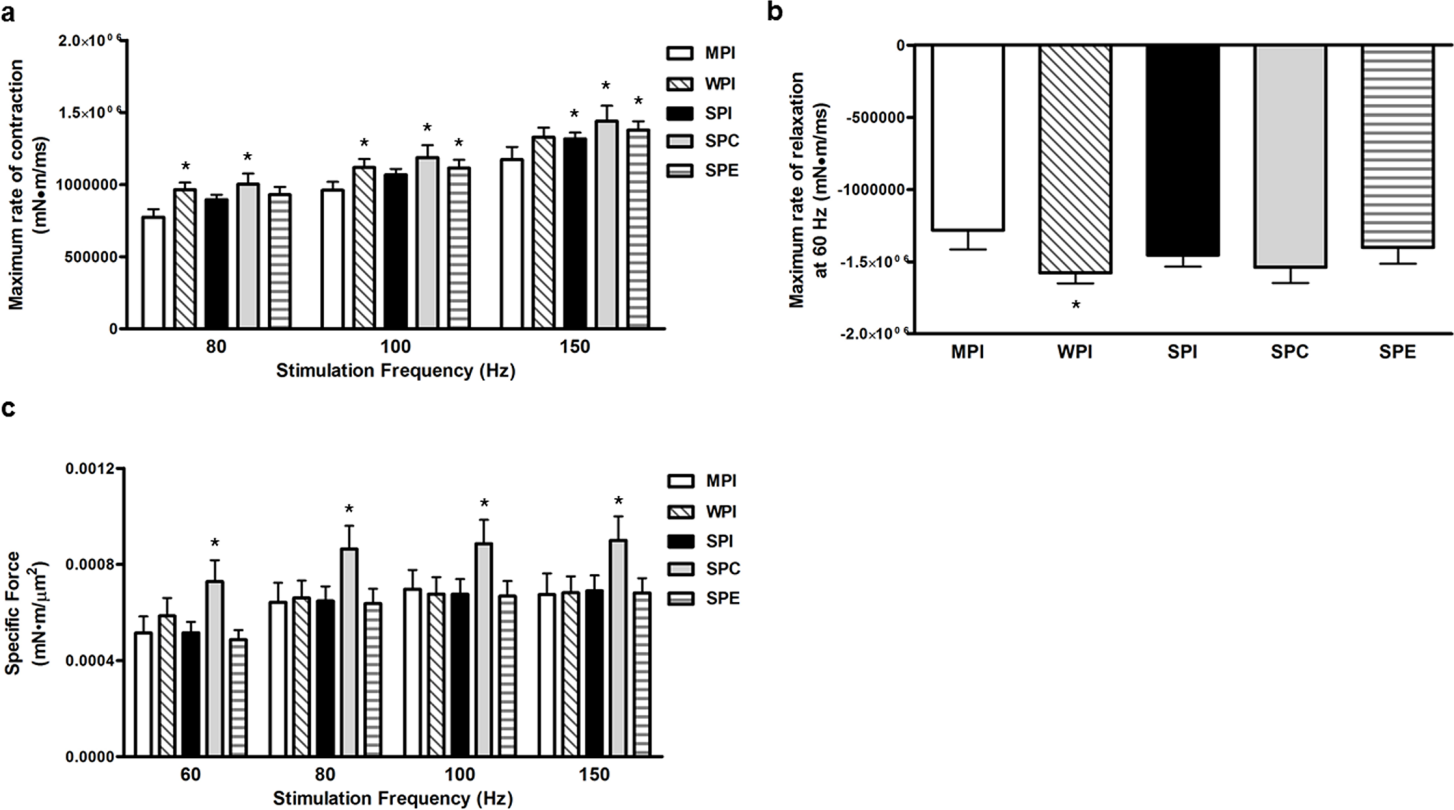
*In vivo* muscle performance at 8 wks. Values are means ± SEM. **a.** Maximum rate of contraction (maximum dF/dt) *in vivo* in the plantarflexor at 8 wks. **b.** Maximum rate of relaxation (minimum dF/dt) *in vivo* in the plantarflexor at 8 weeks. **c.** Specific force (maximum force normalized to total cross-sectional area of the plantarflexor) *in vivo* in the plantarflexor at 8 weeks. *, P<0.05 by Holm-Sidak post hoc analysis compared to MPI group. MPI, milk protein isolate. WPI, whey protein isolate. SPI, soy protein isolate. SPC, soy protein concentrate. SPE, Enzyme-treated soy protein.

Pairwise comparisons show that at 80 Hz, WPI and SPC-fed rats displayed a greater maximum rate of contraction compared to MPI-fed rats (Fig 2A). At 100 Hz, WPI, SPC, and SPE-fed rats also exhibited a greater maximum rate of contraction compared to MPI-fed rats. At 150 Hz, SPI, SPC, and SPE-fed rats displayed a greater maximum rate of contraction compared to MPI-fed rats (Fig 2A). At 60 Hz, the WPI-fed rats displayed a greater rate of relaxation compared to the MPI-fed rats (Fig 2B).

#### Normalized force

Specific force was determined by dividing the maximum force generated at a given stimulation frequency by the total cross-sectional area of the plantarflexor. Specific force was significantly increased with SPC at 60 Hz, 80 Hz, 100 Hz, and 150 Hz compared to MPI (Fig 2C).

#### Force-frequency relationship

The force-frequency relationship was determined by force at any given stimulation frequency normalized to the force produced at 150 Hz for both *in vivo* and *in situ* muscle performance. There were no differences among the groups compared to MPI. (S2 Fig).

#### Fatigue

There were no differences in percent drop in isometric force among the 5 dietary groups after 5 minutes of contractions (S1B Fig).

### End-point measures of muscle performance *in situ*

**Fig 3:**
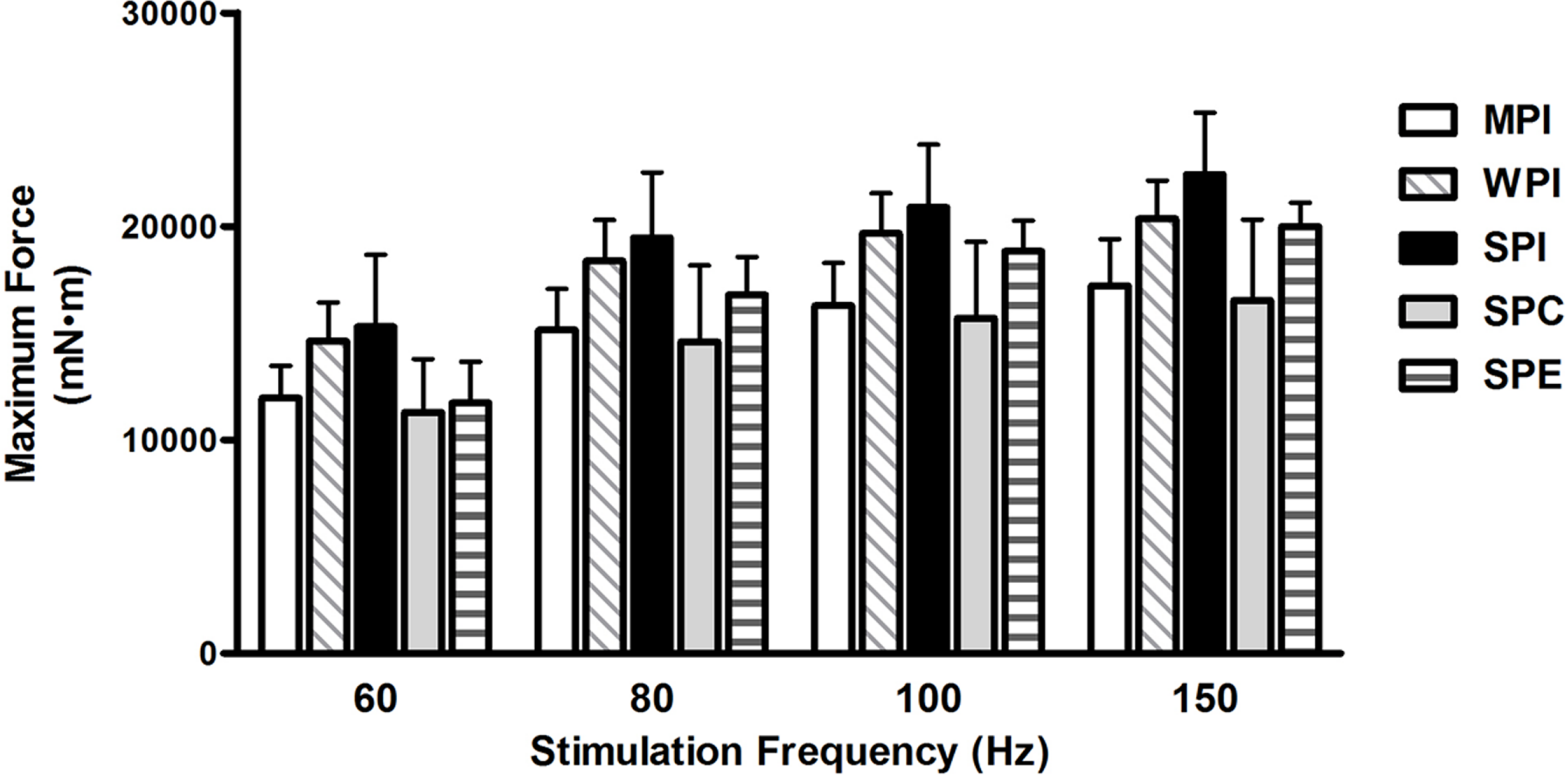
*In situ* muscle performance at 8 wks. Values are means ± SEM. Maximum force *in situ* measured at 8 weeks.

There was no effect of diet among the 5 groups for muscle performance as assessed by maximum force. (Fig 3). There was also no effect of diet on maximum rate of contraction or maximum rate of relaxation.

### End-point measures of muscle morphology and fiber type

**Fig 4:**
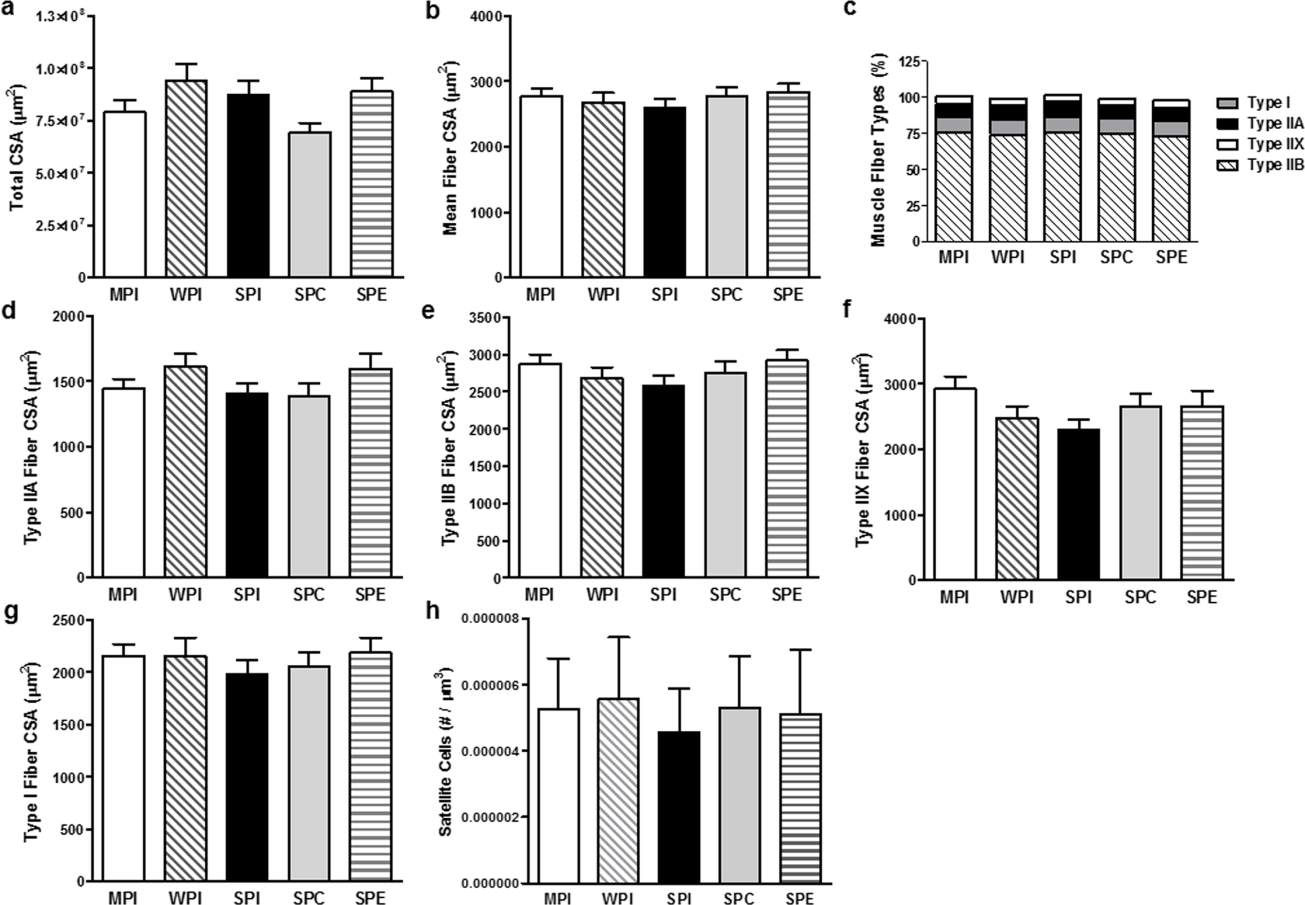
Muscle fiber cross-sectional area. Values are means ± SEM **a.** Total cross-sectional area (CSA) in the plantarflexor. **b.** Mean muscle fiber CSA in the plantarflexor. **c.** Percentages of muscle fiber types in the plantarflexor. **d.** Type IIA muscle fiber CSA in the plantarflexor. **e.** Type IIB muscle fiber CSA in the plantarflexor muscle. **f.** Type IIX muscle fiber CSA in the plantarflexor muscle. **g.** Type I muscle fiber CSA in the plantarflexor muscle. **h.** Satellite cell composition in the plantarflexor muscle. There were no significant differences between groups for any of the measures.

Total cross-sectional area of the plantarflexor muscles (gastrocnemius, soleus, and plantaris) was not different between diet treatments (Fig 4A). This was echoed by the lack of difference in the mean cross-sectional area of all of the fiber types due to diet treatment (Fig 4B). The percentage cross-sectional area of the various muscle fiber types (type I, type IIA, type IIB, and type IIX) were not altered by diet treatment (Fig 4C–4G). There were also no differences in number of satellite cells due to diet treatments (Fig 4H). Representative images can be found in S3 Fig.

## Discussion

In this study, we determined whether plant vs. animal protein source affects skeletal muscle mass and its function. We addressed this question in adult mature rodents on the backdrop of a typical “Western” diet, with high fat and high carbohydrate content. We found that soy protein, processed as an isolate or concentrate, or enzymatically treated, is superior to milk protein isolate in its ability to promote improved muscle function without adversely affecting muscle resistance to fatigue. We show that after 8 weeks of supplementation, SPI, SPC, SPE supplementation resulted in improved contractile kinetics without changes in muscle weight, fiber size, or fiber type. Additionally, SPC increased force production *in vivo* at frequencies above 60 Hz when compared to MPI, and SPI, SPC, and SPE improved force production *in vivo* at 150 Hz. To our knowledge, this is the first study to show that soy protein has direct benefits on muscle function in the context of Western diet in mature rats when compared to total milk protein.

While MPI is a common protein source, WPI is widely considered as the gold standard in protein supplementation and is commonly used by athletes to improve muscle function [9]. Here, we compared MPI and WPI to soy protein supplementation. Both WPI and SPC resulted in similar increases in muscle contractility, supporting SPC as an adequate source of protein similar to the gold standard WPI. These findings agree with results from other studies comparing whey and soy protein on myofibrillar protein synthesis, redox status and mTOR activation, both in rodents and humans [10–14]. Surprisingly, although whey has a higher branched-chain amino acid (BCAA) content than soy, both diets had similar benefits on performance, suggesting that once a threshold dose of BCAA is achieved, additional supplementation provides no further benefit [11, 15].

In this study, we used both *in vivo* and *in situ* methods to assay muscle function. The most physiologically relevant is *in vivo* where no dissection is performed, neuro-vascular function is fully preserved, and muscle torque generated by the plantar flexor group (i.e., gastrocnemius, plantaris) is measured across the joint. Using this assay, SPI was shown to be superior to MPI at stimulation frequencies of 100 Hz and 150 Hz. Our measures of plantarflexor function *in vivo* showed increased force production at 150 Hz with SPI, but also with SPC and SPE at 150 Hz. While *in situ* measures provide a more direct assessment of the muscle function, they come at the cost of a partial dissection of the muscle and severing and attaching the tendon to a force transducer. We also cannot exclude that possibility that dissection somehow negatively affects the muscle function assessment in preparations where the force was greater, namely in the WPI and SPC groups. Indeed, given that the *in situ* force measurement requires the tendon to be sutured and tied to the force transducer, the large forces in these groups (as indicated by the *in vivo* testing) may have impacted this preparation.

Interestingly, WPI, SPI and SPC all exerted their effects on force production independent of changes in muscle weight, total cross-sectional area or mean fiber area. Additional, we saw no differences in fiber types or myostatin concentrations across groups, implying that both whey and soy protein increased the quality of the contraction rather than simply increasing its magnitude through size. Similarly, given that there were no differences in the force-frequency relationship, we can exclude differences in innervation or excitability as contributors to the increased force. Given the different composition of whey versus soy protein preparations, we cannot exclude that these two sources of protein act with different mechanisms to improve force production. Indeed, previous work has shown that a blend of whey, caseinate and soy can acutely stimulate muscle protein synthesis better than either protein source alone [11]. Further studies are needed to investigate if an additional benefit can be extracted from combining WPI with SPC. A recent study in humans demonstrated that there was no difference between protein supplementation with a whey protein isolate or soy isolate-whey isolate blend on muscle growth or function in healthy young men undergoing a 12-week resistance-exercise regimen [16]. It appears that in human subjects consuming a protein-replete diet, additional protein supplementation with any type of protein may not add additional benefit on muscle adaptation to resistance training. In the current rat study, the rats were not exercised, so it remains to be determined whether different protein sources affect muscle growth or function in humans not participating in resistance exercise. Results from a study by Beavers et al. [17] suggests that maintenance of lean body mass in subjects enrolled in a weight loss study (with no exercise intervention) was not different between subjects consuming a soy-enriched versus whey or egg protein-enriched diet, but effects on muscle function were not assessed.

A common, if unfounded concern, with soy protein supplementation is the potential for pseudo-estrogen effects of isoflavones, such as genistein, present in soy-derived products, on muscle function. Indeed, one study in resistance trained young adults has shown that soy protein does appear to partially blunt the rise in serum testosterone and attenuates the decrease in elevated cortisol acutely following exercise [18]. However, this study did not demonstrate any differences in exercise capacity between the different supplementation groups. Conversely, genistein or soy supplementation has been shown to provide several benefits *in vivo* or *in vitro* on muscle, including preventing atrophy [19, 20], modulating metabolism [21, 22], and promoting regeneration [23, 24]. Here, we show that SPE, SPC and SPI consumption led to increased muscle function compared with MPI, did not display any detrimental effects and were equivalent in efficacy to WPI. Therefore, soy protein and its components have overall salutary effects on muscle function at the levels administered in this study.

In conclusion, we have shown that soy protein consumption in the context of a high-fat high-carbohydrate diet results in improve muscle function as assayed by force produced when compared with milk protein. The level of benefit of soy protein was equivalent to those afforded by whey protein supplementation and independent of changes in muscle mass or fiber cross-sectional area. Future studies should determine whether whey-soy protein blends result in further improvement in muscle function.

## Supporting information

S1 Fig Muscle fatigue measurement. Isometric force was determined by delivering a series of 100 tetanic stimulations (80 Hz, 0.2 ms pulse, 250 ms duration) at 0.3 Hz frequency for 5 minutes. Fatigue was assessed by determining the fraction of force produced by the initial contraction for each subsequent contraction. This was performed at baseline (a) and following 8 weeks of dietary treatment (b). There were no differences in the drop in isometric force at baseline or after 8 weeks of dietary treatment.
(TIF)

S2 Fig Force-frequency at 8 weeks. The force-frequency relationship was determined by force at any given stimulation frequency normalized to the force produced at 150 Hz for both *in vivo* (a) and *in situ* (b) muscle performance. There were no differences among the groups compared to MPI.
(TIF)

S3 Fig Representative histological images. Fiber typing images for MPI (a), WPI (b), SPI (c), SPC (d), and SPE (e).
(TIF)

S1 Table Body weight over time (g). (PDF)

S2 Table Serum creatinine, lipids and myostatin. (PDF)

S3 Table Terminal organ and muscle weights (g). (PDF)
