# Supplementary figures and images for: Chronic dietary supplementation with soy protein improves muscle function in rats
Source: PLoS One. 2017 Dec 7;12(12):e0189246. doi: 10.1371/journal.pone.0189246 (PMC5720789; doi:10.1371/journal.pone.0189246)

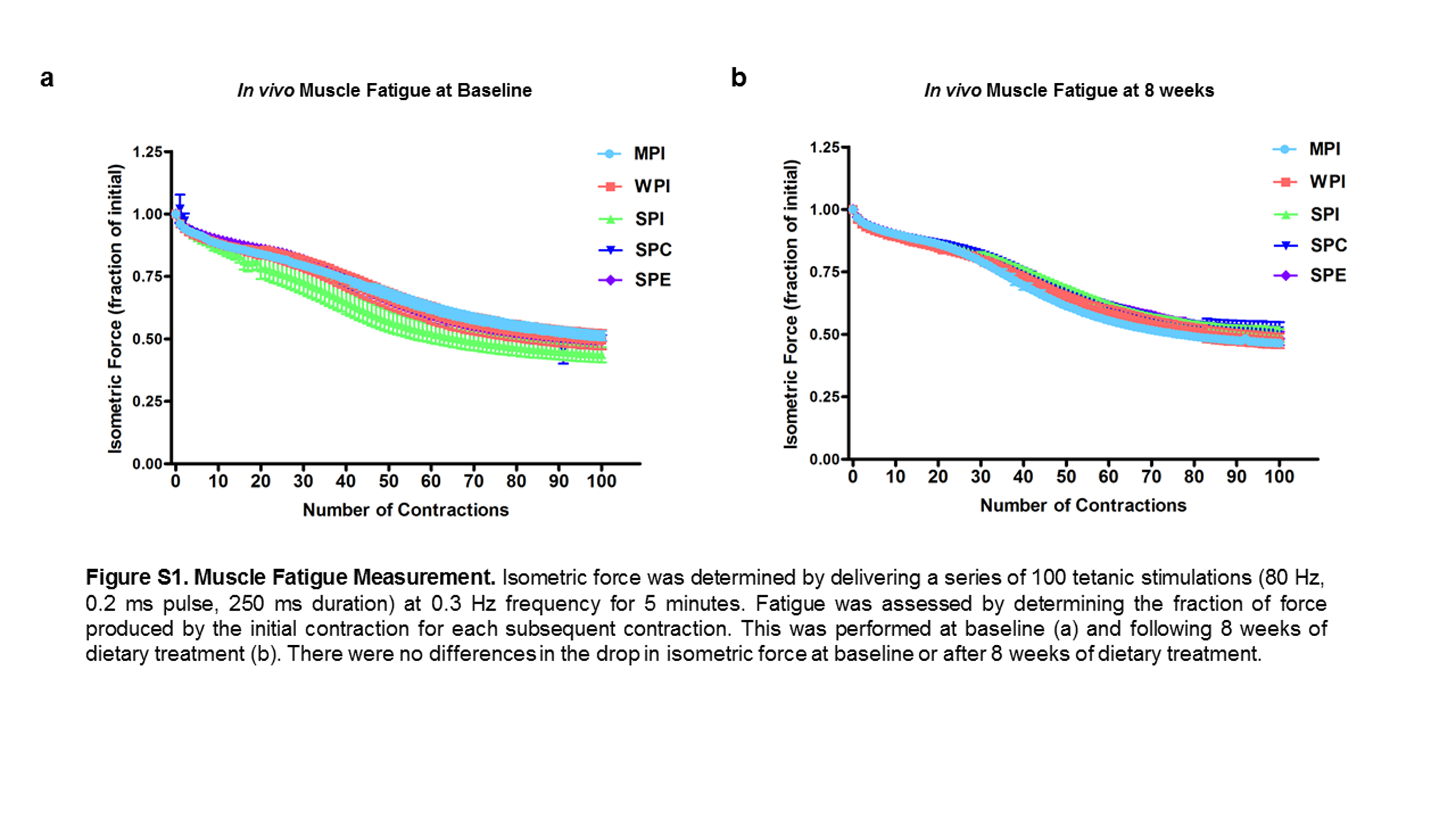

Supplement: S1 Fig — Isometric force was determined by delivering a series of 100 tetanic stimulations (80 Hz, 0.2 ms pulse, 250 ms duration) at 0.3 Hz frequency for 5 minutes. Fatigue was assessed by determining the fraction of force produced by the initial contraction for each subsequent contraction. This was performed at baseline (a) and following 8 weeks of dietary treatment (b). There were no differences in the drop in isometric force at baseline or after 8 weeks of dietary treatment. (TIF) [file pone.0189246.s001.tif]

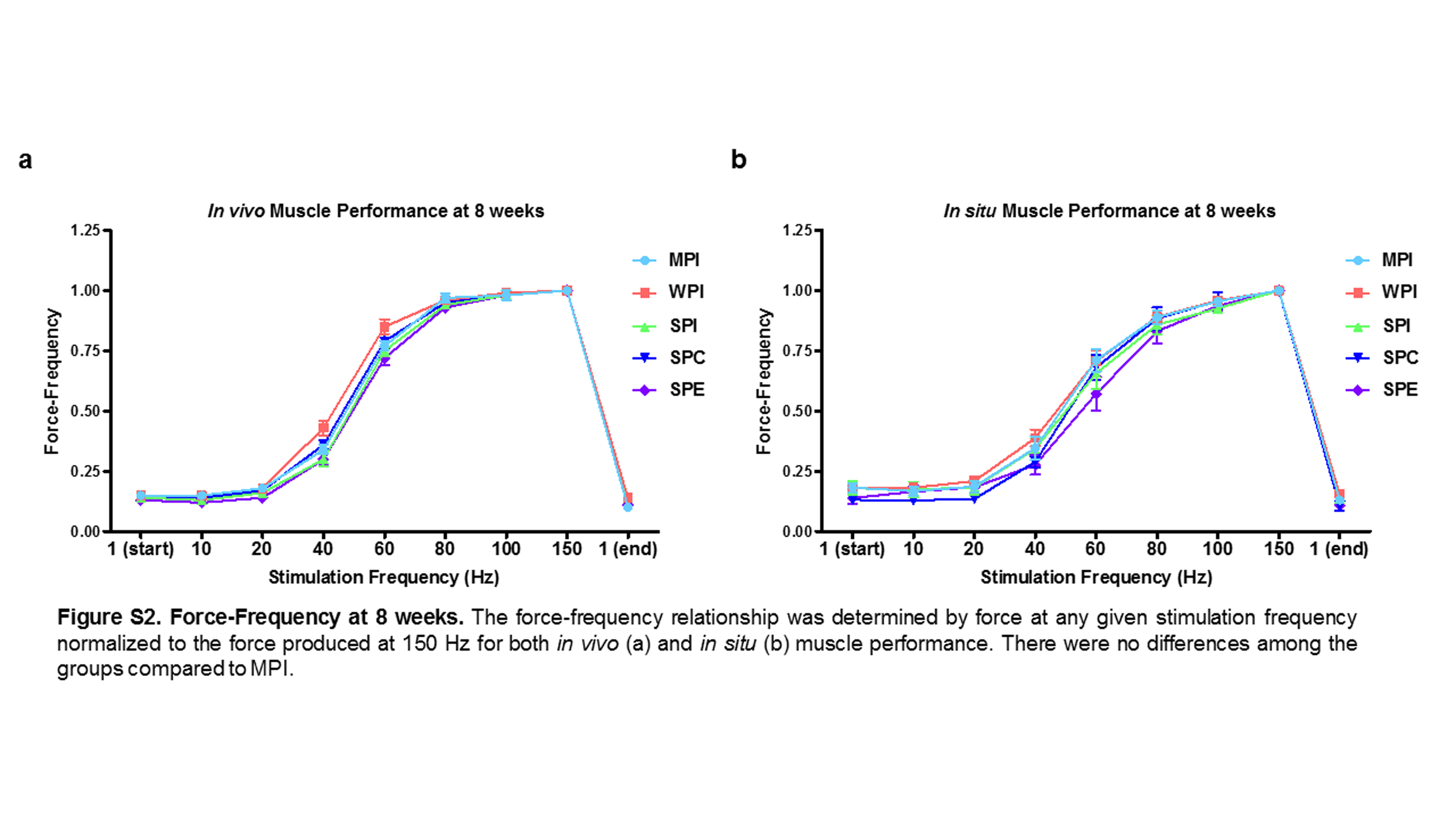

Supplement: S2 Fig — The force-frequency relationship was determined by force at any given stimulation frequency normalized to the force produced at 150 Hz for both in vivo (a) and in situ (b) muscle performance. There were no differences among the groups compared to MPI. (TIF) [file pone.0189246.s002.tif]

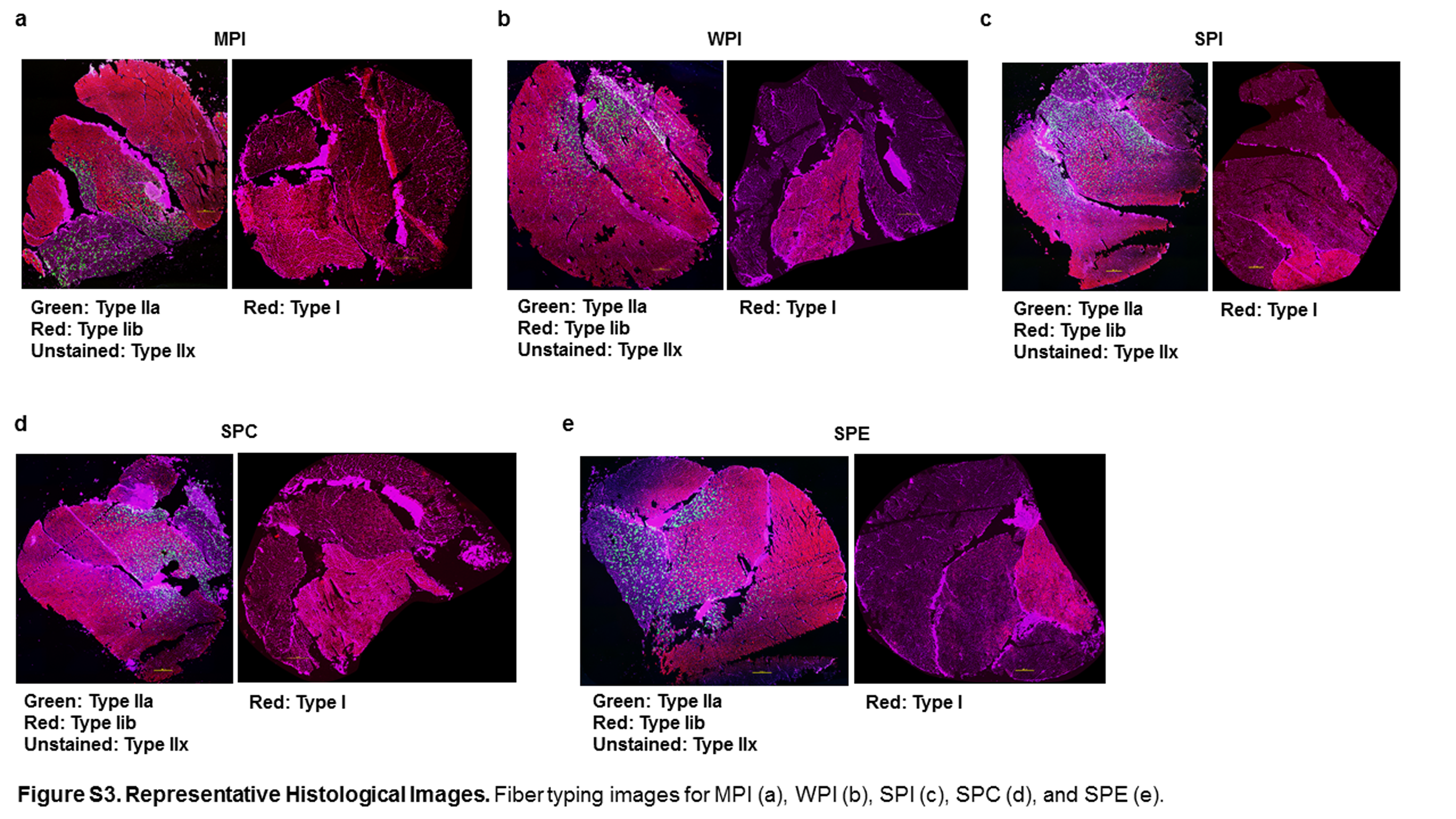

Supplement: S3 Fig — Fiber typing images for MPI (a), WPI (b), SPI (c), SPC (d), and SPE (e). (TIF) [file pone.0189246.s003.tif]
