# Supplementary material for: Chronic dietary supplementation with soy protein improves muscle function in rats
Source: PLoS One. 2017 Dec 7;12(12):e0189246. doi: 10.1371/journal.pone.0189246 (PMC5720789; doi:10.1371/journal.pone.0189246)
Supplement: S1 Table — (PDF) [file pone.0189246.s004.pdf]

S1 Table. Body Weight over Time (g)

Day	Day 12	Day 19	Day 26	Day 33	Day 47	Day 54	Day 61	Day 68	Terminal
**MPI**	474 ± 5	509 ± 7	554 ± 8	593 ± 10	639 ± 20	679 ± 16	704 ± 17	725 ± 19	756 ± 21
**WPI**	473 ± 5	506 ± 7	558 ± 8	605 ± 10	673 ± 14	702 ± 15	727 ± 16	752 ± 18	791 ± 20
**SPI**	472 ± 7	509 ± 9	560 ± 12	604 ± 15	665 ± 17	695 ± 21	719 ± 24	745 ± 27	777 ± 34
**SPC**	469 ± 7	507 ± 10	557 ± 13	600 ± 14	660 ± 19	687 ± 22	712 ± 24	723 ± 24	754 ± 29
**SPE**	477 ± 4	514 ± 5	564 ± 6	611 ± 8	674 ± 9	706 ± 12	728 ± 14	757 ± 15	784 ± 16

Values are means ± SEM. One-way ANOVA analyses were conducted and there were no differences between groups at any time point.
