# Supplementary material for: Chronic dietary supplementation with soy protein improves muscle function in rats
Source: PLoS One. 2017 Dec 7;12(12):e0189246. doi: 10.1371/journal.pone.0189246 (PMC5720789; doi:10.1371/journal.pone.0189246)
Supplement: S2 Table — (PDF) [file pone.0189246.s005.pdf]

S2 Table. Serum Creatinine, Lipids and Myostatin

	mg/dL			pg/mL
	Creatinine	Serum TG	Serum Chol	Myostatin
**MPI**	0.21 ± 0.07	138.4 ± 57.1	91.6 ± 16.9	7546 ± 464
**WPI**	0.21 ± 0.07	181.5 ± 108.4	81.0 ± 14.5	9811 ± 1926
**SPI**	0.23 ± 0.04	141.8 ± 66.0	78.9 ± 7.2	7738 ± 577
**SPC**	0.23 ± 0.07	116.4 ± 40.1	80.4 ± 6.3	7486 ± 494
**SPE**	0.20 ± 0.12	191.8 ± 94.3	89.2 ± 36.3	9724 ± 3075

Values are means ± SEM. One-way ANOVA analyses were conducted and there were no differences between groups.
