# Supplementary material for: Chronic dietary supplementation with soy protein improves muscle function in rats
Source: PLoS One. 2017 Dec 7;12(12):e0189246. doi: 10.1371/journal.pone.0189246 (PMC5720789; doi:10.1371/journal.pone.0189246)
Supplement: S3 Table — (PDF) [file pone.0189246.s006.pdf]

S3 Table. Terminal Organ and Muscle Weights (g)

	MPI		WPI		SPI		SPC		SPE	
**Heart**	1.83	± 0.11	1.83	± 0.05	1.79	± 0.10	1.83	± 0.06	1.82	± 0.06
**Liver**	21.55	± 1.19	22.79	± 1.27	22.50	± 1.57	20.22	± 0.85	21.52	± 0.76
**Suprarenal Fat**	4.88	± 0.64	5.42	± 0.64	5.34	± 0.62	4.58	± 0.43	5.08	± 0.52
**Epididymal Fat**	17.07	± 1.17	21.38	± 1.79	19.82	± 2.15	20.07	± 2.07	19.52	± 1.37
**Retroperitoneal Fat**	21.43	± 2.10	24.48	± 2.19	23.28	± 2.59	23.67	± 1.81	21.75	± 1.77
**Gastrocnemius**	4.02	± 0.10	3.94	± 0.11	3.76	± 0.13	4.03	± 0.13	4.20	± 0.17
**Tricep**	2.43	± 0.11	2.21	± 0.14	2.34	± 0.15	2.21	± 0.08	2.49	± 0.19
**Quadriceps**	4.55	± 0.29	3.91	± 0.36	3.92	± 0.09	4.42	± 0.20	4.46	± 0.29
**Soleus**	0.33	± 0.01	0.33	± 0.01	0.32	± 0.02	0.34	± 0.01	0.32	± 0.01
**Tibialis Anterior**	1.20	± 0.07	1.26	± 0.07	1.20	± 0.05	1.17	± 0.05	1.20	± 0.07
**EDL**	0.92	± 0.05	0.82	± 0.06	0.92	± 0.06	0.81	± 0.05	0.87	± 0.09

Values are means ± SEM. One-way ANOVA analyses were conducted and there were no differences between groups.
